# Asthma exacerbation and proximity of residence to major roads: a population-based matched case-control study among the pediatric Medicaid population in Detroit, Michigan

**DOI:** 10.1186/1476-069X-10-34

**Published:** 2011-04-23

**Authors:** Shi Li, Stuart Batterman, Elizabeth Wasilevich, Huda Elasaad, Robert Wahl, Bhramar Mukherjee

**Affiliations:** 1 Department of Biostatistics, School of Public Health, University of Michigan, Ann Arbor, MI, USA; 2 Department of Environmental Health Sciences, School of Public Health, University of Michigan, Ann Arbor, MI, USA; 3 Michigan Department of Community Health, Lansing, MI, USA

## Abstract

**Background:**

The relationship between asthma and traffic-related pollutants has received considerable attention. The use of individual-level exposure measures, such as residence location or proximity to emission sources, may avoid ecological biases.

**Method:**

This study focused on the pediatric Medicaid population in Detroit, MI, a high-risk population for asthma-related events. A population-based matched case-control analysis was used to investigate associations between acute asthma outcomes and proximity of residence to major roads, including freeways. Asthma cases were identified as all children who made at least one asthma claim, including inpatient and emergency department visits, during the three-year study period, 2004-06. Individually matched controls were randomly selected from the rest of the Medicaid population on the basis of non-respiratory related illness. We used conditional logistic regression with distance as both categorical and continuous variables, and examined non-linear relationships with distance using polynomial splines. The conditional logistic regression models were then extended by considering multiple asthma states (based on the frequency of acute asthma outcomes) using polychotomous conditional logistic regression.

**Results:**

Asthma events were associated with proximity to primary roads with an odds ratio of 0.97 (95% CI: 0.94, 0.99) for a 1 km increase in distance using conditional logistic regression, implying that asthma events are less likely as the distance between the residence and a primary road increases. Similar relationships and effect sizes were found using polychotomous conditional logistic regression. Another plausible exposure metric, a reduced form response surface model that represents atmospheric dispersion of pollutants from roads, was not associated under that exposure model.

**Conclusions:**

There is moderately strong evidence of elevated risk of asthma close to major roads based on the results obtained in this population-based matched case-control study.

## Background

Asthma is a common inflammatory disorder of the airways characterized by variable and recurring symptoms and airflow obstruction, including attacks of wheezing, shortness of breath, chest tightness, and coughing [1,2]. Asthma causes a significant burden in children and is the principal reason for preventable pediatric hospitalizations. Asthma morbidity has been associated with exposure to several ambient air pollutants, e.g., sulfur dioxide (SO_2_), particulate matter (PM_2.5_) and nitrogen dioxide (NO_2_) [3-7], and recently its relationship to traffic-related pollutants has received considerable attention. These associations have been derived largely in ecological studies employing a variety of techniques, e.g., time-series studies using generalized linear models (GLMs) and generalized additive models (GAMs) [3,4,8], and case-crossover studies using conditional logistic regression models (CLRMs) [5,6,9,10]. Such studies assume that point measurements of ambient air pollutant concentrations provide representative and unbiased exposure measures.

The use of individual exposure measures, e.g., based on residence location or the proximity to emission sources that elevate exposures to pollutants, can avoid the problem of ecological bias. Such studies have been used since the 1990s to investigate risks around point sources of environmental pollution, e.g., incinerators and power plants [11-14]. Wakefield and Elliott [15] discussed the statistical framework for both individual and area-level studies. Diggle et al. [16] described an extension to the parametric modeling framework in Diggle et al. [11] and Diggle et al. [13]. They considered matched case-control designs and used a conditional likelihood approach with a non-linear family of risk functions to study the association of asthma and chronic obstructive airways disease with the proximity of residence to major roads in East London.

Asthma severity is frequently grouped into three classifications on the basis of pulmonary function tests and the frequency of symptoms: well controlled (symptoms ≤2 times per week); not well controlled (symptoms >2 times per week but not daily); and very poorly controlled (symptoms throughout the day) [17]. These classifications were developed largely to guide treatment, although they also help to denote the range of impairment suffered by asthmatics. Additionally, multilevel classifications of disease categories or severity permit the use polychotomous conditional logistic regression (PCLR) [18] in matched case-control analyses, which are more efficient than carrying out separate CLRs for each subgroup. The PCLR analysis is appropriate for matched studies without any ordering or nominal disease classification. Mukherjee et al. [19] considered cases having multiple disease states with a natural ordering in matched case-control studies by using conditional adjacent-category logistic regression (ACLR) models in an analysis of low birth weight in newborns.

The present study describes a population-based matched case-control analysis investigating associations between acute asthma outcomes and proximity of residence to main roads. Asthma cases are grouped into multiple disease categories, based on the frequency of acute asthma outcomes, a proxy for asthma control. We used CLR with distance as both categorical and continuous variables, and also with spline terms for distance. These models are then extended by considering multiple disease states using PCLR models, and the use of a more comprehensive exposure metric using a non-linear function representing a reduced form response surface (RFRS) transformation of the proximity to main roads [20].

## Materials and methods

### Study population and health data

We examined the pediatric population in Detroit, MI served by Medicaid. Medicaid claims data provide the most complete and readily available source of healthcare utilization across Detroit. The population consists mainly of African American children from lower income families, and is considered a high risk population for asthma-related events [21]. African Americans are disproportionally affected by asthma and have greater morbidity compared to other races, even after controlling for socio-economic status [22]. We identified all children less than eighteen years of age enrolled in Medicaid and residing in a Detroit zip code in the study period, 2004 through 2006. The extracted data, which were obtained from the Michigan Department of Community Health, included an encrypted Medicaid identifier, age, sex, race/ethnicity, utilization dates and diagnostic codes for inpatient admissions and emergency department visits, and geo-coded home residence at the time of each health care visit. To ensure a full claims history, the study population was restricted to those with continuous Medicaid enrollment (no less than eleven months in each year), full Medicaid coverage, and no other insurance.

Asthma cases were identified as all children who made at least one asthma claim during the three-year study period, indicated by primary diagnostic code 493.X (International Classification of Diseases, 9th Revision, Clinical Modification, ICD-9-CM). Individually matched controls were randomly selected from the rest of the Medicaid population on the basis of non-respiratory related illness (poisoning and injury). Controls were defined children with at least one in-patient admission or emergency department visit where the primary diagnosis was injury or poisoning. Each asthma case was initially matched with two controls based on gender, race/ethnicity, and age (within two years). Individuals who had multiple geo-coded coordinates over the study period, indicating that the child had moved residence, were excluded. Deletion of a case or both controls in a matched set due to moving entailed exclusion of the entire stratum, whereas moving of one control led to 1:1 matched strata. A Chi-square test was used to check whether the likelihood of moving was associated with case-control status in the initial data set. The data were summarized in the form of counts and percents across gender, race and age groups by case-control status.

### Distance measurements

The geo-coded residence information was used to estimate the direction and distance to major roads in Detroit, defined as state and interstate freeways and major arterials with annual average daily traffic (AADT) flows exceeding 50,000 and 20,000 vehicles per day, respectively. The freeways included I75, I94, I96, M10 and M39; the arterials included 8 Mile, Michigan Avenue, Gratiot, Grand River, Fort St., Warren, Mack and Greenfield. In this paper, we call the freeways "primary roads" and the arterials "secondary roads." Shape files providing coordinates of road centerlines were obtained from the Southeast Michigan Council of Governments (SEMCOG). These files and the geo-coded claim data were merged in ARCGIS 9.3 Desktop Software and the "Near" function was used to determine the proximity to each major road.

Several factors affect the accuracy of the distance estimates. Due to confidentiality concerns, claim locations were reported only to the closest 10 m, while the claim location itself is typically a property parcel that spans 20 or 25 m in extent. The road centerline does not account for the width of the highway and median strip, if any, which can exceed 30 m for sections of some freeways. Taken together, these factors suggest that differences on the order of at least 20 to 50 m will be meaningful.

The matched case-control dataset contained a wider range of distances around major roads (up to 6,000 m) as compared with previous studies. We performed a sensitivity analysis while restricting the study region within 1,000 m buffer of primary roads. As before, each asthma case was matched with a random control (within 1,000 m) by gender, race/ethnicity, and age (within 2 years).

### Statistical models

In an individually matched case-control study, effects of potential risk factors are ascertained through conditional likelihood approach, typically using CLR. The residence distance from roads was considered as both categorical (e.g., two-level factor: <300 m or >300 m; three-level factor: 0-200 m, 200-500 m and 500 m or more) and continuous variables. In both cases, because the matching of the potential risk factors of gender, race and age, CLRs were fitted using the distance exposure as the only covariate, i.e.,(1)

where *Y*_*ij *_and *distance*_*ij *_are the case-control status and the distance exposure for the *j*-th individual in the *i*-th stratum, respectively; and *γ*_*i *_is the nuisance parameter from stratum. Without loss of generality, we can specify the first subject in each stratum as the case, and the corresponding conditional likelihood for model (1) is then:

Usually, the parameter estimates *β*_1 _in model (1) are realized by maximizing the above conditional likelihood, which has eliminated the nuisance parameter *γ*_*i*_. In the following, we write the models in an unconditional form with the understanding that parameter estimates and inference are based on the corresponding conditional likelihood.

Model (1) was compared with the null model of no relation by the Wald test of *H*_0 _: *β*_1 _= 0. In this model, a linear relationship between logarithm of the odds and distance was assumed when using continuous distance as the covariate. As a further exploratory step, we also used a spline term corresponding to distance under the conditional likelihood framework [23,24]. A more comprehensive exposure based on the distance to major roads is the predicted concentrations from the RFRS, which closely matched the traffic related concentration profile and fits exposure for each wind angle and downwind distance [20]. The model is given by(2)

where *RFRS(distance*_*ij*_*) *is the reduced form response surface predicted exposure at *distance*_*ij*_; *k*_*1*_*, k*_*2 *_and *k*_*3 *_are the fitted coefficients representing the scale, off-set and decay for the first exponential decay; and *k*_*4*_*, k*_*5 *_and *k*_*6 *_are similar coefficients for the second exponential decay. The two exponential terms represent fast and slow decay processes, which portray dispersion and dilution processes governing emissions and airborne concentrations from "line" sources such as roadways. To avoid use of complex non-linear regression routines, we used the parameters estimated by Batterman et al. [20], i.e. *k*_*1 *_= 0.3087, *k*_*2 *_= 9.8362, *k*_*3 *_= 0.0037, k_4 _= *0.9062, k*_*5 *_= 6.7156, *k*_*6 *_*= *0.0317. *RFRS(distance*_*ij*_*) *is now a fixed transformation of distance and can be treated as a surrogate exposure in a regression model. Linear and the spline term corresponding to this proxy exposure measure were also examined.

We then extended these models by considering cases that had multiple disease states. The asthma cases were further categorized into two subclasses: individuals making only 1 claim (*Y*_*ij *_= 1); and those making 2 or more claims (*Y*_*ij *_= 2). This classification was used as the ordinal response in the PCLR model [18], which is given by(3)

The corresponding conditional likelihood for model (3) is:

where *k*_*i *_is the disease status of the case subject in the *i-*th matched set. Therefore, the parameter estimates and inference of (*β*_11_,*β*_12_) can be realized by performing separate CLRs for each disease group. We also examined the estimated spline terms of distance *spline*_*k*_*(distance*_*ij*_*)*, RFRS transformation of distance *RFRS(distance*_*ij*_), and estimated spline terms of RFRS transformed distance *spline*_*k*_*(RFRS(distance*_*ij*_)) in the PCLR model setting (3).

## Results

### Descriptive analyses

**Figure 1:**
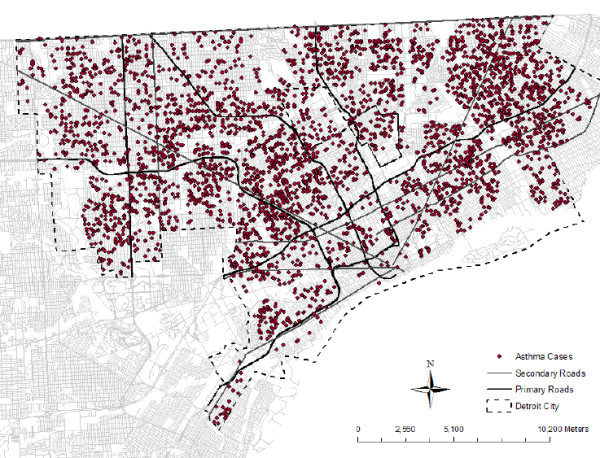
**Primary and secondary roads in the Detroit city area with asthma cases shown as dots**.

Figure 1 shows the primary and secondary roads in the Detroit city area, along with the locations of asthma cases (as dots). Characteristics of cases and controls, summarized in the form of counts and percent distribution across gender, race, age groups, and distance to road, are presented by case-control status in Table 1. The likelihood of moving during the 3 year study period (indicated by multiple geocoded coordinates) was not associated with being a case or control (p = 0.53). Overall, 13% of individuals had multiple geocoded coordinates and were excluded, leaving 5,338 cases, matched with 9,308 controls. Cases and controls were predominantly male, African American, and 0 to 5 years of age. On average, cases lived slightly closer to primary roads, although this difference was not statistically significant from the Wilcoxon rank-sum test (p = 0.15). Distance variables were rounded to integer values and set to 20 m for values less than 20 m.

**Table 1:** Characteristics of cases and controls for the population-based matched case-control dataset from the Pediatric Medicaid population in Detroit, Michigan, 2004-2006.

Characteristics	Case	Control	Total
Number of subjects	5338	9308	14646 (100%)

Gender			

Male	3176^1 ^(59.5%)^2^	5500 (59.1%)	8676 (59.2%)

Female	2162 (40.5%)	3808 (40.9%)	5970 (40.8%)

Race			

Caucasian	224 (4.2%)	408 (4.4%)	632 (4.3%)

African American	4966 (91.9%)	8493 (91.2%)	13459 (91.9%)

Indian	1 (<0.1%)	1 (<0.1%)	2 (<0.1%)

Other/Unknown	34 (0.6%)	85 (0.9%)	119 (0.8%)

Hispanic	113 (2.1%)	322 (3.5%)	435 (2.9%)

Age(years)			

0-5	2782 (52.1%)	4751 (51.0%)	7533 (51.4%)

6-10	1388 (26.0%)	2490 (26.8%)	3878 (26.5%)

11-17	1168 (21.9%)	2067 (22.2%)	3235 (22.1%)

Distance from primary road (m)	1386.4^3 ^(20-5847) ^4^	1437.0 (20-5951)	1418.7 (20-5951)

Distance from secondary road (m)	1342.2 (20-5099)	1347.2 (20-5129)	1344.5 (20-5129)

^1 ^Count

^2 ^Percentage

^3 ^Mean

^4 ^Range

Characteristics of patients in the population-based matched case-control data set of asthma from the pediatric Medicaid population in Detroit, Michigan, 2004-2006. The data were summarized in the form of counts and percents across gender, race, and age groups, and in the form of mean and ranges of distance from major roads by case-control status.

Of the 5,338 cases, 66.2% had 1 asthma claim, 15.7% had 2 claims, and 18.1% had 3 or more claims during the study period (Additional file 1, Figure S1). A few individuals made a much larger number of claims over the study period, e.g., one case experienced 34 claims. We categorized cases into two groups, those making only 1 claim and those making 2 or more claims, and later used this classification as the ordinal response in the PCLR models.

### Conditional logistic regression models

**Figure 2:**
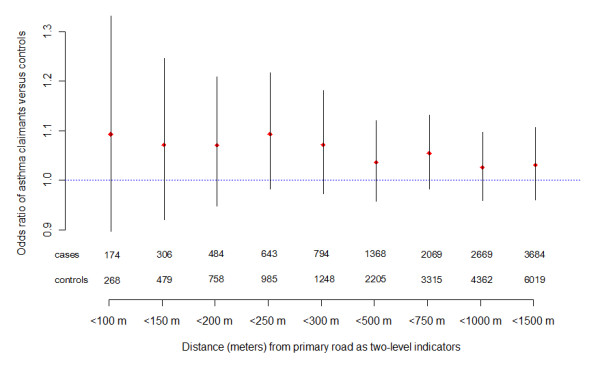
**Estimated odds ratios with 95% confidence intervals of asthma for distance thresholds from primary roads**. Estimated odds ratios with 95% confidence intervals of asthma for different distance thresholds from primary road, as well as the number of subjects lying in the two level indicator of distance from primary road by the case-control status. Each estimate is based on conditional logistic regression results using distance as a dichotomous factor indicating residence location inside or outside the corresponding buffer, for the population-based matched case-control data set of asthma from the pediatric Medicaid population in Detroit, MI, 2004-2006.

Figure 2 shows odds ratios (ORs) of being an asthma claimant compared to controls at various distances from a primary road, along with 95% CIs and the number of cases and controls in each buffer. Distances from 100 to 1,500 m were tested using the two-level distance indicator (e.g., <300 m or >300 m). These ORs are suggestive of an association between being an asthma claimant and proximity to main roads, especially at shorter distances (i.e., <300 m), however, statistical significance was not attained. No statistically significant relationship was seen for secondary roads. We also carried out an analysis where instead of multiple two-level models, distance was categorized into seven levels: 0-100 m, 100-250 m, 250-500 m, 500-750 m, 750-1,000 m, 1,000-1,500 m and >1,500 m, with the last buffer used as the reference category. The analysis, presented in the Additional file 1, Figure S2, shows a trend in the ORs, though no categories reached statistical significance. This analysis was then restricted to buffers within 1,000 m of the primary road. This localized sensitivity analysis, showed in the Additional file 1, Figure S3 and S4, indicated more pronounced effects even though the sample size was reduced to 2,669 cases and 2,669 controls. For example, the OR of being an asthma claimant living within 100 m of a primary road was 1.45 (95% CI: 1.14, 1.84); the OR attenuated to 1.20 (95% CI: 1.04, 1.39) at 200 m, and to 1.08 (95% CI: 0.96, 1.21) at 300 m using two-level models. Similar effects were found for these distance buffers in models using distance as a multi-level categorical factor. The OR of an asthma case in 0-100 m and 100-250 m buffers was 1.59 (95% CI: 1.23, 2.06) and 1.21 (95% CI: 1.02, 1.45) respectively, both compared to distances exceeding 750 m (Additional file 1, Figure S4).

Table 2 shows estimated ORs and 95% CIs of being an asthma claimant associated with 1,000 m increase in distance to roads, using CLR models and distance as a linear and continuous variable, as shown in model (1). Three models are shown: primary roads only; secondary roads only; and both types of roads in the same model. Asthma claimants were associated with proximity to primary roads with an estimated OR of 0.97 (95% CI: 0.94, 0.99) for a 1,000 m increase in distance from the primary road; this association remained when primary and secondary roads were both included in the model. The corresponding OR was 0.84 (95% CI: 0.72, 0.98) in the sensitivity analysis of the restricted study region to 1000 m. No association was found using RFRS transformed distance as an argument in model (1). The RFRS measure is very heavily weighted towards very short distances to roadways (below 200 m), and exposures at larger distances are given very little weight. This may be a more realistic exposure surrogate to consider than a linear distance term, although it did not reach significance in this particular study.

**Table 2:** Estimated odds ratios for asthma claims using binary response conditional logistic regression models.

Model	Covariate	Term	**OR**^**1**^	**95% CI**^**2**^		P-value
1	Primary Road	Primary	**0.971**	**0.944**	**0.999**	**0.04**

2	Secondary Road	Secondary	0.995	0.966	1.025	0.74

3	Primary Road + Secondary Road	Primary	**0.970**	**0.944**	**0.998**	**0.04**

		Secondary	0.992	0.963	1.022	0.59

4	RFRS^3^(Primary Road)	Primary	1.059	0.736	1.525	0.75

5	RFRS(Secondary Road)	Secondary	0.983	0.739	1.309	0.90

6	RFRS(Primary Road) +RFRS(Secondary Road)	Primary	1.060	0.736	1.526	0.76

		Secondary	0.983	0.738	1.308	0.90

^1 ^OR: Odds ratio

^2 ^CI: Confidence interval

^3 ^RFRS: Reduced form response surface transformation

Estimated odds ratios for experiencing an asthma claim with a 1,000 m increase in distance from main roads or with a 1-unit increase of RFRS transformed distance, using binary response (cases versus controls with cases defined by any asthma claims recorded in 2004-2006) conditional logistic regression models.

**Figure 3:**
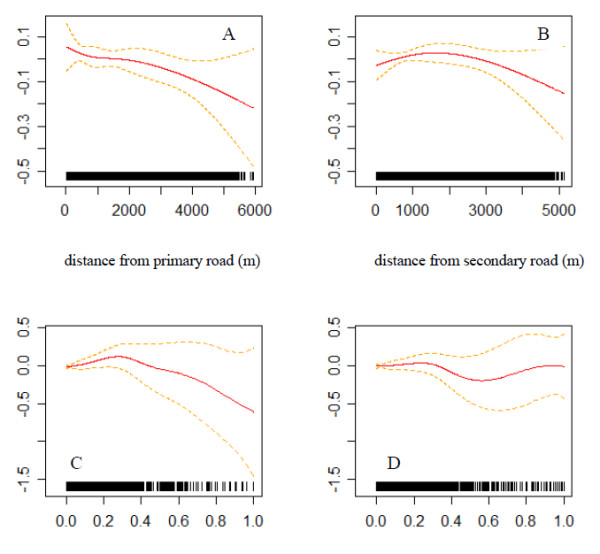
**Estimated natural spline terms of distance and RFRS transformed distance showing the distance-odds relationships**. * RFRS: Reduced form response surface. Estimated natural spline terms of distance and RFRS transformed distance showing the distance-odds relationships for having an asthma claim, using binary response conditional logistic regression models. The solid lines show the point estimates; the dashed lines show the 95% confidence regions.

Figure 3 illustrates the natural spline fit and 95% CI for the relationship between distance to roadway and odds of being an asthma claimant using the CLR model in model (1). The distance-odds ratio relationship appears to be monotonic with risk increasing with proximity to primary roads (Figure 3a). No such relationship is seen for secondary roads. These plots provide a useful look at the data and indicate that a linear distance-odds relationship might be adequate for primary roads.

### Polychotomous conditional logistic regression models

Table 3 shows the estimated odds ratios with 95% CIs of asthma claims associated with 1,000 m increase in distance to the main road, using the PCLR model with distance as a continuous variable. In this model, an additional 1,000 m distance between residence location and primary roads was associated with a reduction of asthma claims with an estimated odds ratio of 0.97 (95% CI: 0.94, 0.99) between cases making exactly 1 claim and controls, and an estimated odds ratio of 0.98 (95% CI: 0.93, 1.04) between cases making 2 or more claims and controls. This association was also found when distances from primary and secondary roads were included together in the PCLR model (3). No association was found using RFRS transformed distance as an argument. These results resemble those from the CLR (shown in Table 2). The direction and effect size for individuals making multiple claims is suggestive of a dose-response relationship; however, statistical significance was not attained, probably a result of the smaller number of individuals making multiple claims as compared with those making single claims.

**Table 3:** Estimated odds ratios for asthma events using polychotomous conditional logistic regression models.

Model	Covariate	Term	Single claimants versus controls				Multiple claimants versus controls			
			**OR**^**1**^	**95% CI**^**2**^		**P-value**	**OR**	**95% CI**		**P-value**

1	Primary Road	Primary	0.982	0.933	1.035	0.50	**0.967**	**0.935**	**0.999**	**0.04**

2	Secondary Road	Secondary	0.979	0.927	1.035	0.46	1.002	0.967	1.037	0.93

3	Primary Road + Secondary Road	Primary	0.980	0.930	1.033	0.45	**0.966**	**0.935**	**0.999**	**0.04**

		Secondary	0.977	0.924	1.033	0.42	0.998	0.963	1.034	0.91

4	RFRS^4^(Primary Road)	Primary	1.131	0.583	2.194	0.72	1.029	0.665	1.593	0.90

5	RFRS(Secondary Road)	Secondary	1.163	0.678	1.994	0.58	0.922	0.658	1.293	0.64

6	RFRS(Primary Road) + RFRS(Secondary Road)	Primary	1.126	0.580	2.184	0.73	1.031	0.666	1.596	0.89

		Secondary	1.160	0.676	1.990	0.59	0.922	0.658	1.292	0.64

^2 ^OR: Odds ratio

^3 ^CI: Confidence interval

^4 ^RFRS: Reduced form response surface transformation

Estimated odds ratios for asthma events with a kilometer increase of distance from main roads or with a 1-unit increase of RFRS transformed distance, using polychotomous conditional logistic regression models with two sub-types of cases: single claims and cases with two or more claims in the study period.

## Discussion

Positive associations of respiratory disorders with traffic-related air pollutants have been reported in several studies [16,25-28]; however, no associations were found to be statistically significant for asthma in these studies. In a case-control (cases = 417, controls = 461) study in Erie County, New York, pediatric (less than fourteen years) hospitalizations for asthma were related to living near a road with heavy traffic, using a CLR adjusted for age and poverty level. Children hospitalized for asthma were more likely to live on roads with the highest tertile of vehicle miles traveled (OR = 1.93, 95% CI: 1.13-3.29) within 200 m, and were more likely to have trucks and trailers passing by within 200 m of their residence (OR = 1.43, 95% CI: 1.03-1.99) compared to controls [29]. A matched case-control study with 1,809 asthma cases (less than nineteen years) living in Perth, Australia associated residential traffic exposure and children's emergency department presentation for asthma. While risk estimates were sensitive to socio-economic gradients and the type of exposure method, the kernel density measure demonstrated a large increase (OR 2.51, 95% CI 2.00 - 3.15) in the risk of asthma emergency department presentation for the high exposure group compared to the low exposure group [30]. A study of a low-income population in San Diego County, California has examined the locations of residences of 5,996 children (less than fourteen years of age) with asthma. The odds ratio of two or more medical visits compared to one visit was almost three times higher (OR = 2.89; 95% CI, 1.07-7.40) for individuals living near high traffic roads, defined as more than 41,000 vehicles/day at the nearest street [31]. A recent study in Lima, Peru associated asthma symptoms among 725 adolescents (thirteen to fifteen years) with proximity to a high-traffic-density avenue in a periurban shantytown. The odds of asthma in households living within 100 m increased by two-fold (p < 0.05) compared to a reference distance of 384 m (estimated using spline function), using a multivariable logistic additive model [32]. More evidence for adverse effects of residential proximity to traffic sources on asthma is discussed by Salam et al. [33]. A comprehensive critical review of the literature on emissions, exposures, and health effects associated with traffic has been compiled by the Health Effects Institute [34].

The categorical distance analysis in the present study does not provide strong statistical evidence of an association, although the ORs indicate a greater chance of an asthma event with closer proximity to primary roads. Previous studies have focused on highly localized (e.g. <300 m from major roadways) effects of traffic-related air pollution on respiratory health. However, only 14% (Figure 2) of all cases and controls lived within 300 m of primary roads in Detroit. The Medicaid asthma claims in Detroit over the study period (2004-2006) in our dataset was complete and contained a larger number of observations (5,338 cases, 9,308 controls) over a much wider range of distances around major roads (up to 6,000 m) as compared with previous studies. We chose to use wider buffers around major roads (i.e., 500 and 1,000 m) in the discrete analysis, which provided more balanced portions of subjects. We did see stronger relationships in both the two-level and the multi-level analyses when the study region was restricted to 1,000 m buffers around primary roads (Additional file 1, Figure S3 and S4).

The continuous distance analysis provides evidence of an association between asthma claims and proximity to primary roads, with an estimated OR of 0.97 (95% CI: 0.94, 0.99) for a 1,000 m increase in distance from primary roads. Previous studies have suggested that concentrations of many traffic-related pollutants fall to background concentrations with a few hundred meters from large roadways. In part, roadway effects on asthma claims are found at large distances due to the limitations of a linear model. Further spline fits to the distance-odds relationship showed a much sharper decay at shorter distances, and results were significant only within a very small buffer, and purely due to chance beyond a short range. Several factors potentially weakened the distance-odds relationship. We did not accounted for the sharp pollutant gradients anticipated near roads, effects of wind direction and other meteorological variables affecting dispersion, roads other than primary and secondary roads, and the specific traffic densities of the roads. We included the RFRS transformation of distance as a partial solution to the sharp pollutant gradients, with the anticipation that this would reduce exposure misclassification. However, the RFRS model and parameter estimates obtained were based on a highly localized (<200 m) data set, which was shown to be not suitable for this Medicaid data (Figure 3). For example, the precision is low when *RFRS(distance) *is larger than 0.7, due to inadequate sample size within the associated 50 m-buffer (*RFRS(50 m) *= *0.7*). No association was found using RFRS-transformed distance as an argument in the sensitivity analysis when the study region was restricted to a 1,000 m buffer around primary roads.

The PCLR models, involving analyses of children with 2 or more claims, suffered from inadequate sample sizes, which may have been a reason for the lack of statistical significance in these analyses (33.8% made two or more claims among asthma cases, Additional file 1, Figure S1). The results from the PCLR models were dominated by cases having exactly 1 asthma claim (Table 3), which had similar effect size and significance level as those given by the CLR models (Table 2).

The proposed method and application in this study have several limitations. Residence location is a construct which may be confounded by socioeconomic status (SES). Income, education and other data related to SES are not available in Medicaid. However, census data suggest that income and education are distributed ecologically in the study region, and the Medicaid-eligible population studied in Detroit is relatively homogeneous. Thus, additional control of SES as a confounder is not anticipated to substantially alter effect estimates linking residence location and proximity to major roads. For the same reason, we do not anticipate differential biases in the SES between cases and controls. Access to accurate household income and other individual-level data for child's beneficiary would strengthen our analysis. In absence of such direct measures, the present findings potentially can suffer from residual confounding attributable to SES. Additionally, asthma control is strongly tied to clinical management, including medication use. While Medicaid information contains some information regarding prescriptions filled, it does not indicate whether they are used or used correctly. The number of prescriptions filled in the 6 month period prior to a visit for cases (or non-visit for controls), for example, might provide some evidence of long term control, medication overuse/rescue, or other information. However, the development of this information would require a significant effort since each subject (case and control) would require its own history based on the event date.

Possible sources of exposure misclassification are also acknowledged. The actual daily locations and commutes of individuals, indoor air quality of individual homes, and smoking behavior of primary caregivers were not taken into account. The use of the specific locations of the homes of children with asthma claims and a full spatial analysis of pollutant covariates may provide further insight into the geographical pattern of asthma cases, but is beyond the scope of the current paper.

Future studies should focus on improving exposure measurements, e.g., estimating or measuring traffic-related pollutants near homes and schools, and including time/activity patterns in the prediction models. Also warranted is further research investigating differential impacts of traffic by genetic and other susceptibility factors, and identifying those specific pollutants that underlie the adverse effects of traffic on asthma [33].

## Conclusions

Based on an examination of all pediatric Medicaid claims in Detroit for asthma over a three year period, there is reasonable evidence of elevated risk of asthma exacerbation among children with asthma who live close to major roadways. Comparable results were obtained using conditional linear regression and polychotomous conditional logistic regression models. PCLR analyses involving children with two or more asthma claims were suggestive of associations with primary roadway proximity, however, ORs obtained in these models were not statistically significant.

## List of abbreviations

AADT: annual average daily traffic; ACLR: conditional adjacent-category logistic regression; CLRM: conditional logistic regression model; CI: confidence interval; GAM: generalized additive models; GLM: generalized linear model; NO_2_: nitrogen dioxide; OR: odds ratio; PCLR: polychotomous conditional logistic regression; PM_2.5_: particulate matter less than 2.5 micrometers in diameter; RFRS: reduced form response surface; SEMCOG: Southeast Michigan Council of Governments; SES: socioeconomic status; SO_2_: sulfur dioxide.

## Competing interests

The authors declare that they have no competing interests.

## Authors' contributions

All authors have read and approved the final manuscript. SL conducted statistical analyses and drafted portions of the article. SB designed the overall study, directed its implementation, wrote portions of the article, and edited the article. EW assisted in study design, developed Medicaid data analysis, and edited the article. HE provided distance measurement and helped draft diagrams. RW helped draft portions of the article. BM designed the study, directed data analyses, provided statistical methodology insight, and helped draft the article.

## Supplementary Material

Additional file 1 **Appendix for supplementary figures**. This additional file contains 5 supplementary figures for the main text. Click here for file
