# Supplementary material for: Asthma exacerbation and proximity of residence to major roads: a population-based matched case-control study among the pediatric Medicaid population in Detroit, Michigan
Source: Environ Health. 2011 Apr 23;10:34. doi: 10.1186/1476-069X-10-34 (PMC3224543; doi:10.1186/1476-069X-10-34)
Supplement: Additional file 1 — Appendix for supplementary figures. This additional file contains 5 supplementary figures for the main text. [file 1476-069X-10-34-S1.PDF]

Appendix: Supplementary Figures

Asthma exacerbation and proximity of residence to major roads: a population-based matched case-control study among the pediatric Medicaid population in Detroit, Michigan

Shi Li, Stuart Batterman, Elizabeth Wasilevich, Huda Elasaad, Robert Wahl, Bhramar Mukherjee

Figure S1. Frequencies of asthma claims made by the 5338 cases.

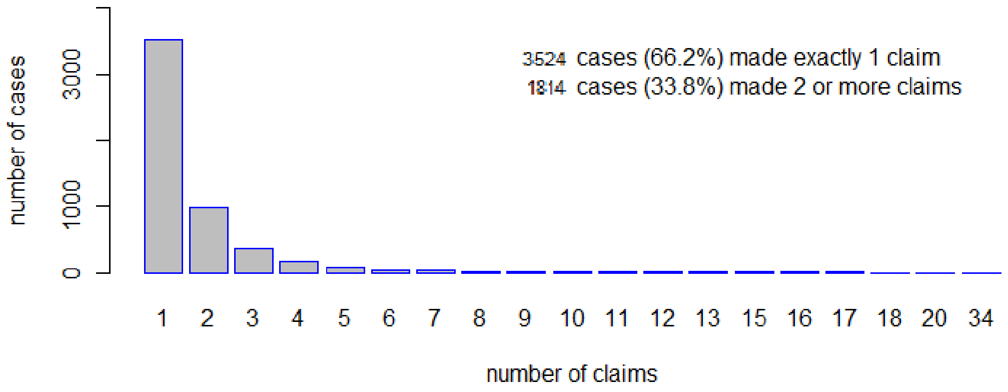

Legend: Frequencies of claims made by the 5338 asthma cases of the population based matched case-control data set from the pediatric Medicaid population in Detroit, Michigan, 2004-2006.

Figure S2. Estimated odds ratios with 95% confidence intervals of asthma for categorized distance from primary road.

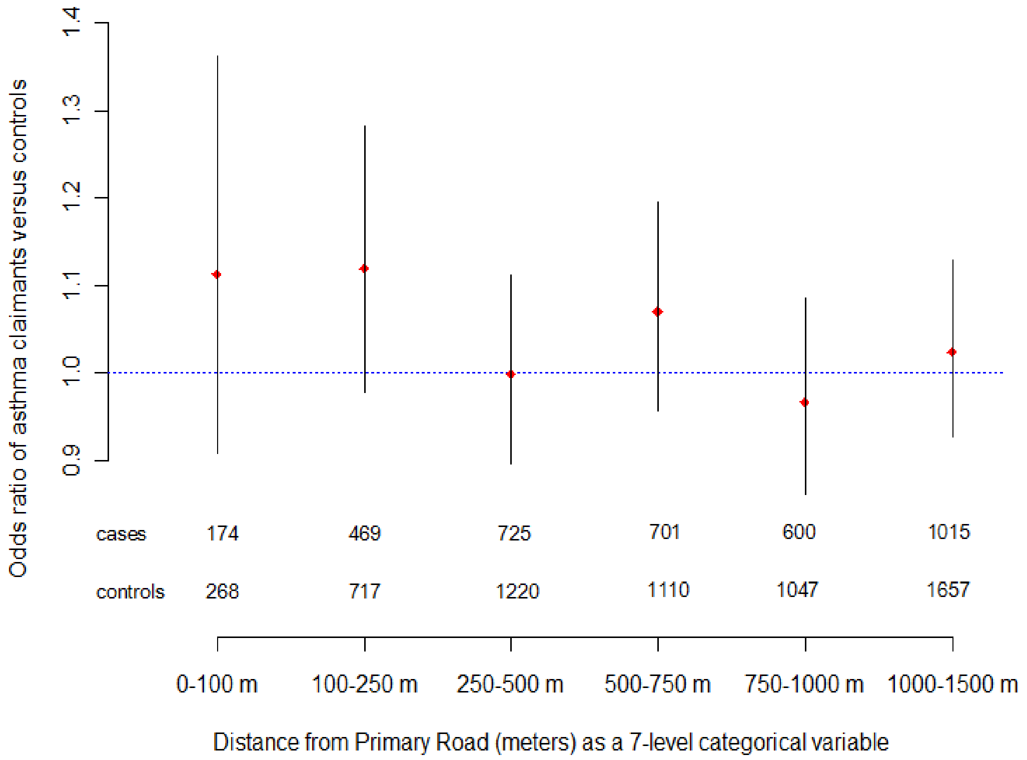

Legend: Estimated odds ratios with 95% confidence intervals of asthma for categorized distance with 7 levels from primary road (reference level 1500 m or more), as well as the number of subjects lying in the exclusive intervals from primary road by the case-control status, for the population-based matched case-control data set of asthma from the pediatric Medicaid population in Detroit, MI, 2004-2006.

Figure S3. Estimated odds ratios with 95% confidence intervals of asthma for distance threshold from primary road, restricting the study region buffer to 1km.

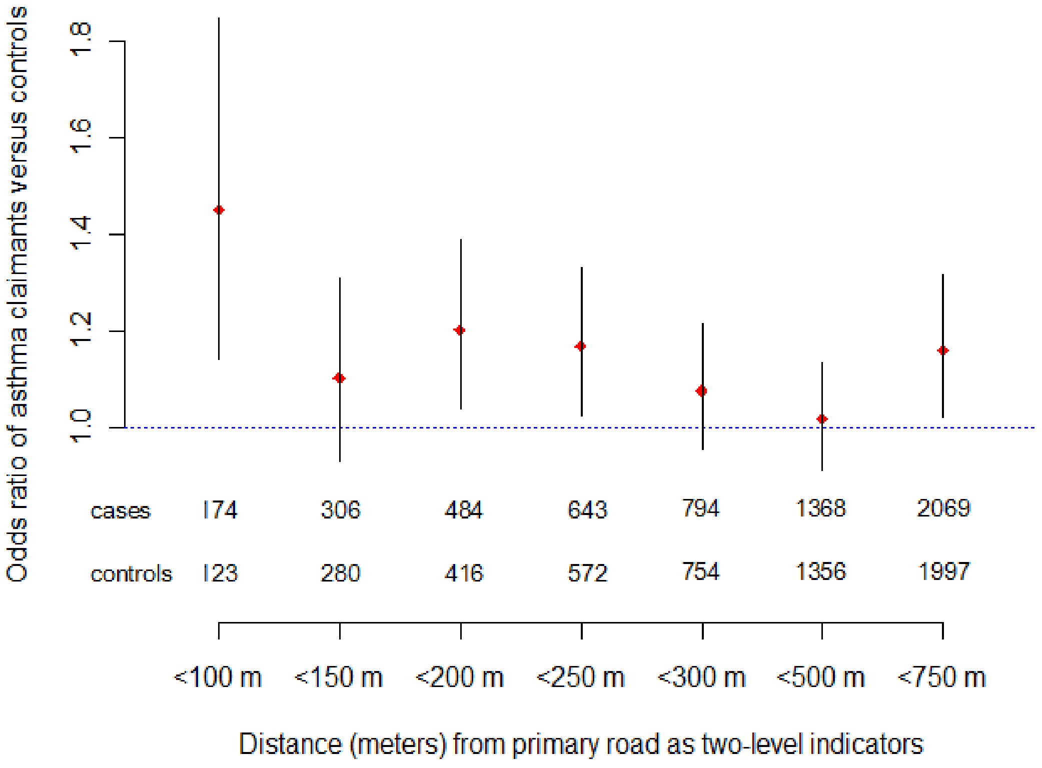

Legend: Estimated odds ratios with 95% confidence intervals of asthma for different distance thresholds from primary road restricting the study region buffer to 1km, as well as the number of subjects lying in the two level indicator of distance from primary road by the case-control status, for the population-based matched case-control data set of asthma from the pediatric Medicaid population in Detroit, MI, 2004-2006.

Figure S4. Estimated odds ratios with 95% confidence intervals of asthma for categorized distance from primary road, restricting the study region buffer to 1km.

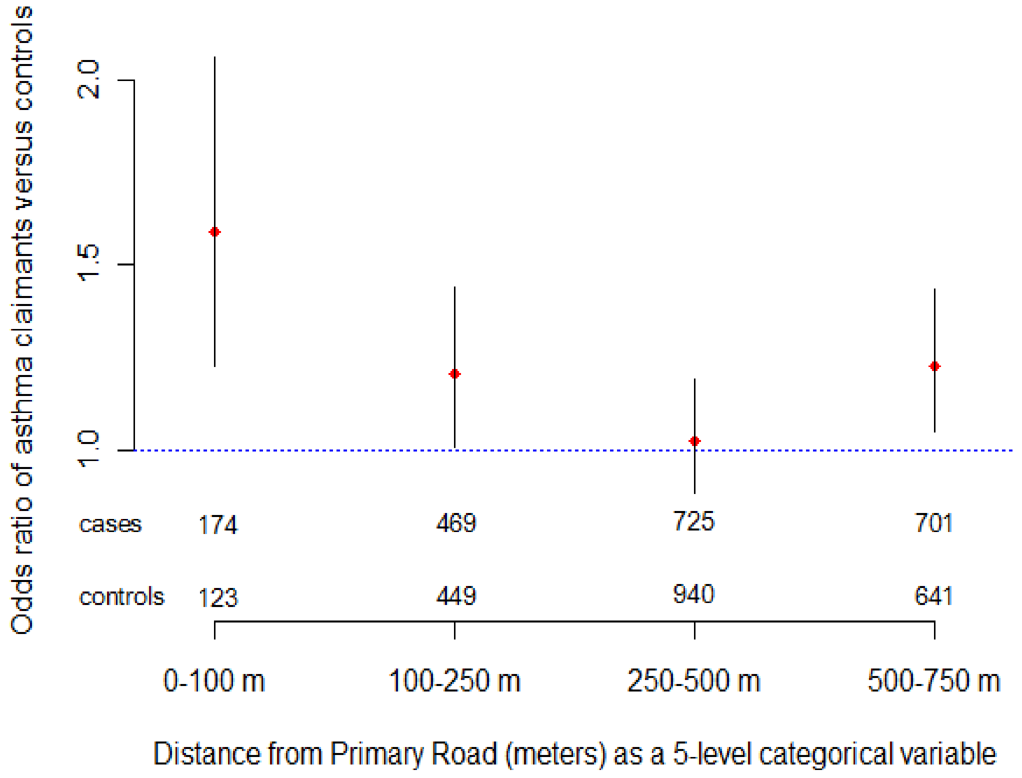

Legend: Estimated odds ratios with 95% confidence intervals of asthma for categorized distance with 5 levels from primary road (reference level 750 m or more), restricting the study region buffer to 1 km, as well as the number of subjects lying in the exclusive intervals from primary road by the case-control status, for the population-based matched case-control data set of asthma from the pediatric Medicaid population in Detroit, MI, 2004-2006.

Figure S5. Estimated natural spline terms of distance showing the distance-odds relationships.

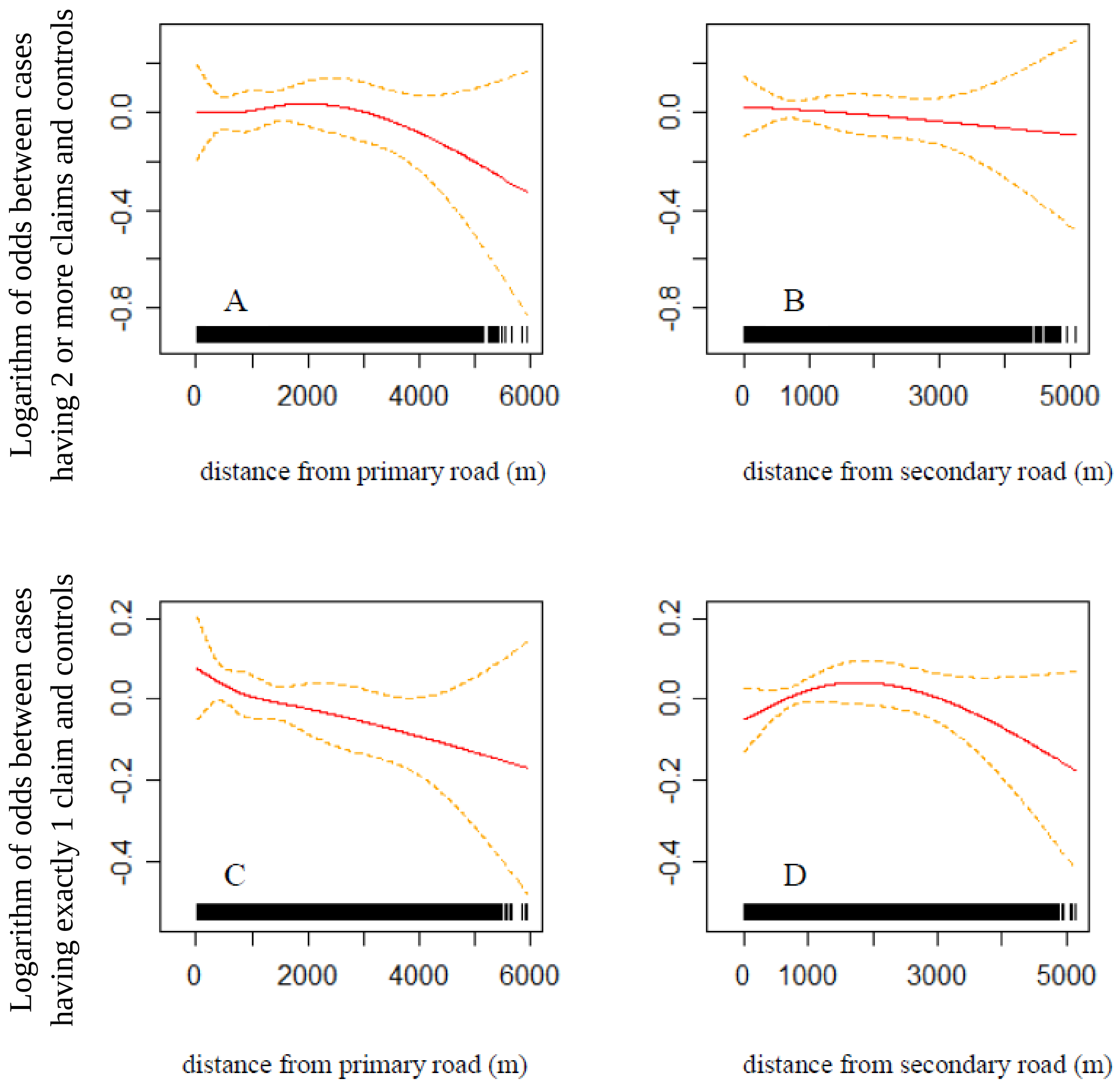

Legend: Estimated natural spline terms of distance showing the distance-odds relationships for asthma using polychotomous conditional logistic regression models. The solid lines show the point estimates; the dashed lines show the 95% confidence regions.
